# Evidence for an Essential Deglycosylation-Independent Activity of PNGase in *Drosophila melanogaster*

**DOI:** 10.1371/journal.pone.0010545

**Published:** 2010-05-10

**Authors:** Yoko Funakoshi, Yuki Negishi, J. Peter Gergen, Junichi Seino, Kumiko Ishii, William J. Lennarz, Ichiro Matsuo, Yukishige Ito, Naoyuki Taniguchi, Tadashi Suzuki

**Affiliations:** 1 Glycometabolome Team, Systems Glycobiology Research Group, RIKEN Advanced Science Institute, Wako, Saitama, Japan; 2 Department of Biochemistry and Cell Biology and the Center for Developmental Genetics, Stony Brook University, Stony Brook, New York, United States of America; 3 Department of Biochemistry and Cell Biology and Institute for Cell and Developmental Biology, Stony Brook University, Stony Brook, New York, United States of America; 4 Department of Chemistry and Chemical Biology, Gunma University, Kiryu, Gunma, Japan; 5 Synthetic Cellular Chemistry Laboratory, RIKEN Advanced Science Institute, Wako, Saitama, Japan; 6 Glycotrilogy Project, Exploratory Research for Advanced Technology (ERATO), Japan Science and Technology Agency (JST), Kawaguchi, Saitama, Japan; 7 Department of Disease Glycomics, The Institute of Scientific and Industrial Research, Osaka University, Ibaraki, Osaka, Japan; 8 Disease Glycomics Team, RIKEN Advanced Science Institute, Wako, Saitama, Japan; 9 Core Research for Evolutionary Science and Technology (CREST), Japan Science and Technology Agency (JST), Kawaguchi, Saitama, Japan; National Institute on Aging, United States of America

## Abstract

**Background:**

Peptide:*N*-glycanase (PNGase) is an enzyme which releases *N*-linked glycans from glycopeptides/glycoproteins. This enzyme plays a role in the ER-associated degradation (ERAD) pathway in yeast and mice, but the biological importance of this activity remains unknown.

**Principal Findings:**

In this study, we characterized the ortholog of cytoplasmic PNGases, PNGase-like (Pngl), in *Drosophila melanogaster*. Pngl was found to have a molecular weight of ∼74K and was mainly localized in the cytosol. Pngl lacks a CXXC motif that is critical for enzymatic activity in other species and accordingly did not appear to possess PNGase activity, though it still retains carbohydrate-binding activity. We generated microdeletions in the *Pngl* locus in order to investigate the functional importance of this protein *in vivo*. Elimination of *Pngl* led to a serious developmental delay or arrest during the larval and pupal stages, and surviving mutant adult males and females were frequently sterile. Most importantly, these phenotypes were rescued by ubiquitous expression of *Pngl*, clearly indicating that those phenotypic consequences were indeed due to the lack of functional *Pngl*. Interestingly, a putative “catalytic-inactive” mutant could not rescue the growth-delay phenotype, indicating that a biochemical activity of this protein is important for its biological function.

**Conclusion:**

Pngl was shown to be inevitable for the proper developmental transition and the biochemical properties other than deglycosylation activity is important for its biological function.

## Introduction

Peptide:*N*-glycanase (PNGase) catalyzes the cleavage of the amide linkage of β-glycosyl asparagine of *N*-linked glycoproteins. PNGase activity was originally discovered in almond emulsion [1] and subsequently in *Flavobacterium meningosepticum* [2]. Since that time this enzyme has been widely used as a powerful reagent for analyzing the structure and functions of *N*-linked glycan chains on glycoproteins. However, the biological significance of this enzyme itself has been undocumented until recently.

The presence of PNGase activity in animals was first reported in embryos of *Oryzias latipes* (Medaka fish) [3] followed by the discovery in various mammalian cell lines [4]. While the fish PNGase was active at acidic pH and therefore was believed to be of lysosomal origin [5], the mammalian PNGase has a neutral pH optimum, and the activity was mainly found in the cytosol [6]. Cytoplasmic PNGase is widely distributed between cells and organs in mammals [4], [7], suggesting that this enzyme is involved in important basic biological processes. A gene encoding the cytoplasmic PNGase (*PNG1*) was identified in *Saccharomyces cerevisiae*, and found to be well-conserved throughout eukaryotes [8], further providing evidence for its biological importance [9], [10].

**Figure 1:**
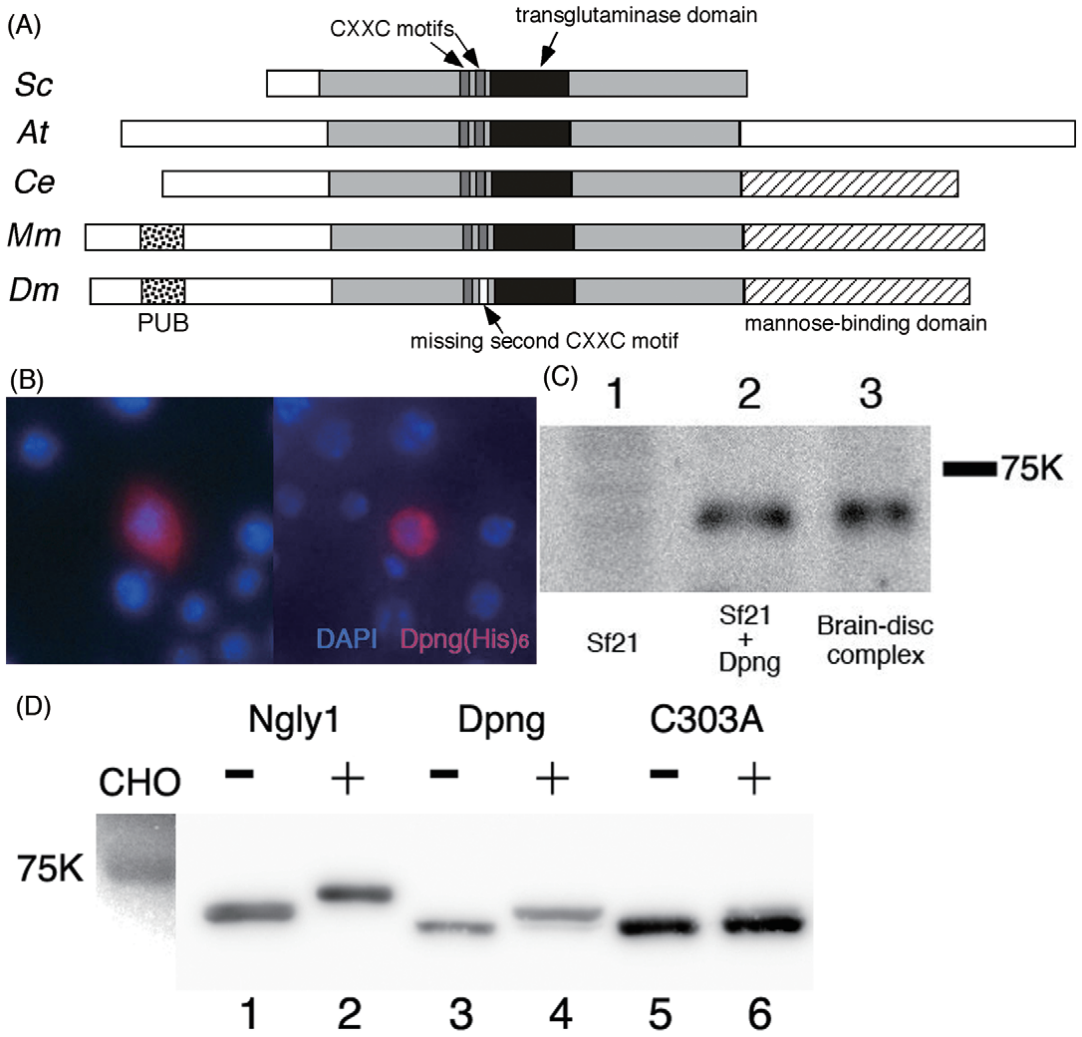
Pngl also conserves characteristics of cytosolic PNGases from other species. (A) Schematic representation of PNGase orthologs from various species. Sc: *Saccharomyces cerevisiae*; At: *Arabidopsis thaliana*; Ce: *Caenorhabditis elegans*; Mm: *Mus musculus*; Dm: *Drosophila melanogaster*; the conserved core domain is colored by light grey. The transglutaminase domain with catalytic center: black, PUB domain: dotted-filled, and mannose-binding domain, filled with hatched lines. Two CXXC motifs are described with thin rectangles colored by dark grey. In the case of Dm, one of the rectangles is colored by white as CXXC is not conserved. (B) Cytosolic staining of (His)_6_-tagged Pngl expressed in *Drosophila* S2 cells, stained by anti-His antibody(red). Blue staining is for nuclear staining (DAPI) (C) Western blotting analysis of PNGase proteins expressed in Sf21 cells. Pngl was immunoprecipitaed with anti-Pngl antibody and was detected by Western blotting as described in “Materials and Methods”. Lane 1: Immunoprecipitated samples from mock-transfected Sf21 cell soluble fraction, lane 2: from Sf21 cells transfected with *Pngl*, lane3: from larval disc-brain-complex soluble fraction. (D) Glycan binding assays of mouse (Ngly1) and fly (Pngl) PNGases. Lanes 1, 3, 5: negative control (no probe) of Ngly1, Pngl, and Pngl(C303A); lanes 2, 4, 6: Ngly1, Pngl, and Pngl(C303A) after reaction with the carbohydrate probe.

From an evolutionary standpoint, cytoplasmic PNGase is an interesting protein with a diverse structural arrangement (Fig. 1A, [9], [10]). The core domain of this enzyme ortholog is highly conserved and due to its homology with transglutaminase and the conservation of amino acids comprising the catalytic domain, cytosolic PNGase has been categorized as a member of transglutaminase superfamily [9], [11].

It has been established that the deglycosylation activity of PNGase is involved in the process called endoplasmic reticulum-associated degradation (ERAD), that is responsible for the degradation of proteins that fail to acquire their correct folding state during protein synthesis [12], [13], [14]. Recently, cytoplasmic PNGase-mediated deglycosylation during the ERAD process was also suggested to be critical for class I major histocompatibility complex antigen presentation [15], [16]. Despite the accumulating knowledge on the role of cytoplasmic PNGase in the ERAD and the conservation of its structural features in species from yeast to mammals [17], [18], [19], [20], [21]), the biological significance of this protein has not yet been rigorously demonstrated in any system. For instance, the null mutant for *png1* in *S. cerevisiae* was shown to have no apparent phenotype [8]. It has been reported that mutation of the PNGase ortholog in *Neurospora crassa*, which was also shown to be inactive for the PNGase activity due to the amino acid change at catalytic triad essential for the deglycosylation activity [18], [22], caused the malformation of hyphae, but the molecular mechanism remains to be determined [23], [24].

In this study, we examined the biological functions of the *Drosophila melanogaster* PNGase ortholog, PNGase-like (Pngl) (registered as PNGase or CG7865 in FLYBASE). As found in other species Pngl was mainly localized in the cytosol. Interestingly, the Pngl lacks a second CXXC motif that is critical for enzymatic activity in other species [18], [22]. Biochemical assays indicate that Pngl retains carbohydrate-binding activity, but lacks conventional PNGase activity, suggesting that the deglycosylation activity may have been lost during the evolutionary process. *Drosophila* mutant for *Pngl* showed severe developmental defects and reduced viability, and the surviving adults are frequently sterile. These phenotypes were reversed by expression of a *UAS-Pngl* transgene, demonstrating that developmental defects were indeed due to the lack of functional Pngl. On the other hand, a “catalytically-inactive” mutant where the Cys in Pngl equivalent to catalytic Cys in other PNGase orthologs was changed to Ala did not rescue the growth-delay phenotype, strongly suggesting that the unknown biochemical activity of Pngl is important for its biological function.

## Results

### Flies have a PNGase ortholog localized in the cytosol

The *Saccharomyces cerevisiae* gene encoding cytoplasmic PNGase was first identified by isolating mutants defective in PNGase activity, followed by genetic mapping of the mutation responsible for the loss of enzyme activity [8]. The fly ortholog of PNGase has the core transglutaminase (PNGase) domain conserved throughout species (Fig. 1A), with an extended N-terminus containing a PUB (Peptide:*N*-glycanase/UBA or UBX-containing proteins) domain, the p97-interacting domain found in the murine protein [25], and a C-terminal putative mannose binding domain [19].

As in the case of PNGases of other species, Pngl does not have any signal peptide, which likely indicates the cytosolic localization. Consistent with this prediction, cytosolic staining of Pngl(His)_6_ was observed (Fig. 1B). It should be noted that part of Pngl could be localized on the ER membrane, as in the case with mouse PNGase ortholog, Ngly1 [26]. Ngly1 was reported to be associated with the ER membrane from the cytosolic face by interacting with other ER resident proteins such as gp78 [27] or Derlin1 [28]. Because of the lack of the antibody acting to the fly ER protein available to us, further detail on its subcellular localization could not be examined. The occurrence of Pngl in the cytosol was also revealed by Western blotting. A specific band consistent with the expected molecular weight of ∼74K was detected with the anti-Pngl antibody in cytosol fraction prepared from Pngl-expressing Sf21 cells (Fig. 1C, lane 2) while no band was detected in the supernatant prepared from mock pVL1393-transfected Sf21 cells (Fig. 1C, lane 1). A similar specific band was detected in the cytosol fraction prepared from Canton S wildtype larvae disc-brain complex. Taken all together, these results indicate Pngl is a cytosolic protein, as is the case for PNGase orthologs in other species [8], [26], [29], [30].

### Pngl conserves carbohydrate binding property

As Pngl conserves many structural features with the other PNGases previously studied, it may be possible that Pngl still retains some of the biochemical characteristics of the cytoplasmic PNGase. For instance, Pngl retains all of the residues critical for the carbohydrate-binding activity of yeast Png1 and mouse Ngly1 [21], [31], [32]. To investigate this possibility, Pngl was expressed in Sf21 cells and the cytosolic fraction was assayed for carbohydrate-binding using the haloacetamide-derivative of high mannose-type *N*-glycan probe that covalently binds to the catalytic Cys residue of ScPng1 (C303 in Pngl) [31]. It is important to note that we did not detect PNGase activity in the cytosolic fraction of native Sf21 cells (data not shown). A shift in migration similar to that obtained with FLAG-tagged Ngly1 (Fig. 1D, lanes 1, 2) was observed for FLAG-tagged Pngl after reaction with the carbohydrate probe (Fig. 1D, lanes 3 and 4). The specificity of the covalent bond-formation with the glycan probe was confirmed by the fact that the binding was abrogated by conversion of the reactive Cys303 to Ala (Fig. 1D, lanes 5 and 6). This result clearly indicates that the binding of probe to the wild-type Pngl was specific to Cys303 and not due to the result of non-specific binding to other Cys residues on this protein. It is therefore concluded that the *Drosophila* ortholog of PNGase retains carbohydrate-binding activity as in the case of other cytosolic PNGases.

### Pngl does not possess deglycosylation activity

**Figure 2:**
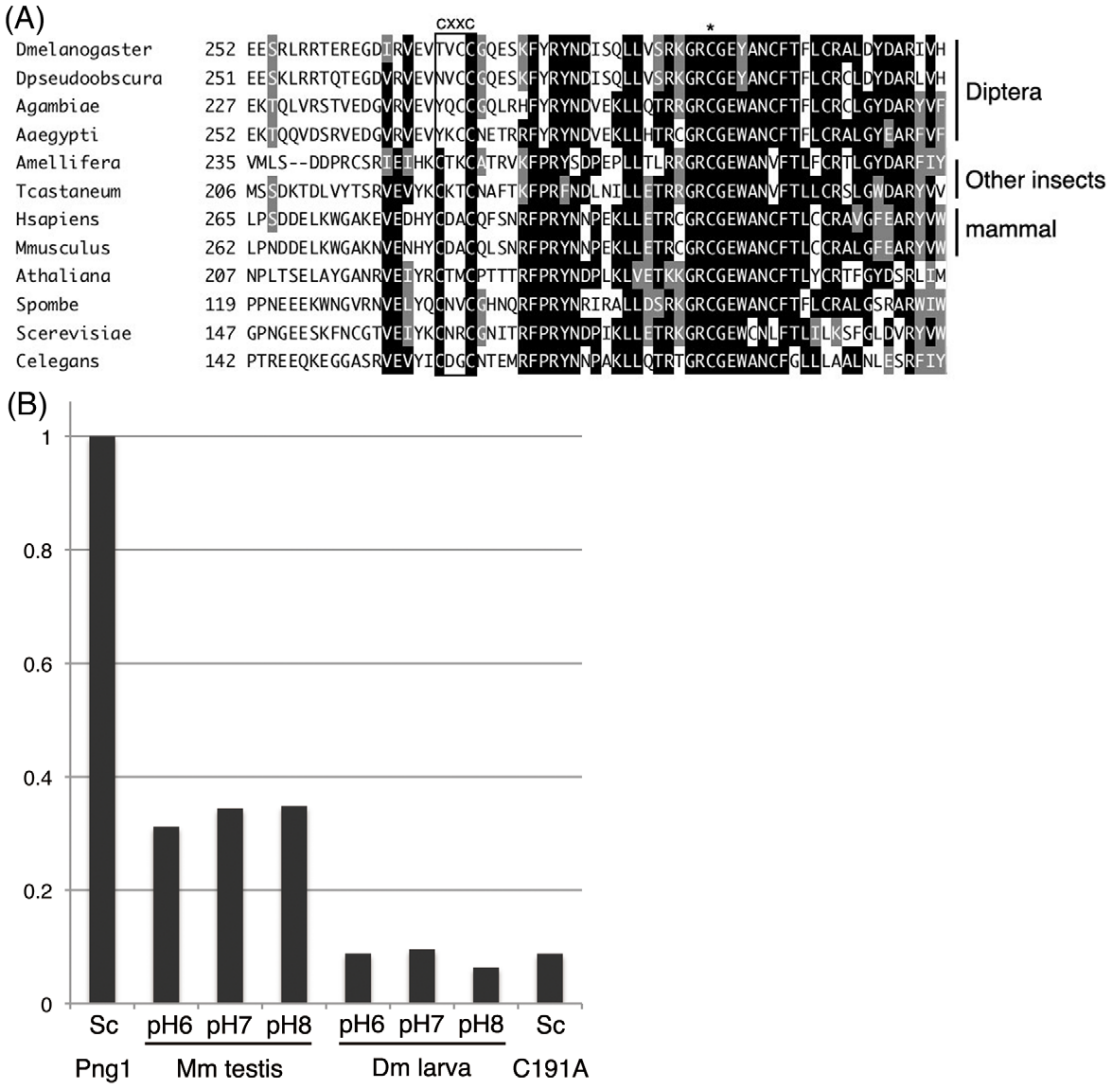
Loss of PNGase activity due to the lack of CXXC motif. (A) Cytosolic PNGase orthologs from Diptera do not conserve zinc-binding motif. Amino acid comparison of the region around one of the CXXC motifs (boxed), critical for PNGase activity; the abbreviations used are: Dmelanogaster, *Drosophila melanogaster*; Dp, *Drosophila pseudoobscura*; Aaegypti, *Aedea aegypti*; Agambiae, *Anopheres gambiae*; Amellifera, *Apis mellifera*; Tcastaneum, *Tribollium castaneum*; Hsapiens, *Homo sapiens*; Mmuscalus, *Mus muscalus*; Athaliana, *Arabidopsis thaliana*; Spombe, *Schizosaccharomyces pombe*; Scerevisiae, *Saccharomyces cerevisiae*; and Celegans, *Caebinohabiditis elegans*. Conserved C (Cys) in the catalytic triad is shown by the asterisk. (B) Lack of PNGase activities in the soluble fractions of whole larval homogenate. PNGase activities were examined with the ^14^C-labeled asialofetuin glycopeptides as a substrate. The relative activity of the yeast ScPng1 was set to 1. ScPng1, wild type Png1 from *Saccharomyces cerevisiae* recombinant protein (positive control); Mm testis, testis from *Mus muscalus* homogenate; Dm larva, *Drosophila melanogaster* 3rd instar wandering larvae homogenate; pH6, pH6.7 (optimal pH for ScPng1 [8]); pH7, pH7.4 (optimal pH for mouse Ngly1 [6]); pH8.3 (optimal pH for CePNG1 from *C. elegans* [39]); Sc C191A, C191A mutant recombinant of ScPng1 (inactive control). Activity observed with ScPng1 C191A mutant can be regarded as background.

In spite of high homology with the overall structure especially with mammalian PNGase, whose deglycosylation activity is already reported [6], [7] and indeed some characteristics are conserved as described before, there is still a notable structural feature of Pngl, i.e. the absence of a second CXXC motif close to the catalytic transglutaminase domain that is found in other PNGase orthologs (Fig. 1A). The lack of this motif was also observed in other insects belonging to Diptera, suggesting that the second CXXC motif might have been lost during evolution (Fig. 2). The CXXC motif was shown to have a zinc binding activity, and was suggested to maintain a stable conformation of the catalytic transglutaminase domain that is required for deglycosylation activity [18], [22]. This observation raises the possibility that Pngl does not have deglycosylation activity, despite the high degree of conservation in the catalytic domain (61% identity; 75% similarity in the transglutaminase domain). Consistent with this, we failed to detect deglycosylation activity in the soluble fractions prepared from larval homogenate (Fig. 2B) or recombinant Pngl protein expressed in Sf21 cells (data not shown) under various conditions. Furthermore, analysis of free oligosaccharides (fOSs), one of the possible reaction products by PNGase, accumulated in wild type and *Pngl* mutant did not exhibit the significant difference, thus failing to provide credible evidence for its PNGase activity (Text S1 and Table S1). This result contrasted with the case of budding yeast, where fOSs were virtually absent in *png1Δ* strain [33], [34]. In mammalian cells, the cytosolic PNGase-independent formation of fOSs has well been documented, while its molecular mechanism remains to be clarified [35], [36]. It is therefore reasonable to assume that the flies are capable of generating fOSs even with the loss of PNGase activity in Pngl.

To determine if amino acid differences in the second CXXC motif of Pngl are responsible for the loss of deglycosylating activity, we took advantage of a PNGase assay using the yeast Png1 (ScPng1)-expression system in *E. coli* [37]. Amino acids of the second CXXC motif (amino acids 165–168) of yeast Png1 were replaced by amino acids found in the equivalent position of Pngl and the recombinant proteins were assayed for activity. As shown in Table 1, the ScPng1 mutants were enzymatically active as long as they retained the CXXC motif (ex. ScPng1(CVCC)), but became inactive if the motif was disrupted with the amino acids of fly ortholog (TVCC), which correspond to the CXXC motif (ex. ScPng1(TNRC); ScPng1(TVCC)). The finding that even the single amino acid substitution of the Cys165 of ScPng1 to the Thr (ScPng1(TNRC)) abrogates enzyme activity strongly suggests that Pngl has lost its deglycosylation activity due to mutation of the zinc binding motif during the evolutional process.

**Table 1:** Expression and activity of various CXXC mutants of ScPng1.

	The second CXXC motif	Relative activity (%)
WT	CNRC	1.0
N166V, R167C	CVCC	0.79
C165T	TNRC	n.d.
C165T, N166V, R167C	TVCC	n.d.
C191A (inactive)	CNRC	n.d.

Relative activity was calculated by setting wild type activity as 1.0 and inactive mutant as 0.0.

n.d.: not detected (below background level).

### Expression levels and patterns of *Pngl* are developmentally regulated

**Figure 3:**
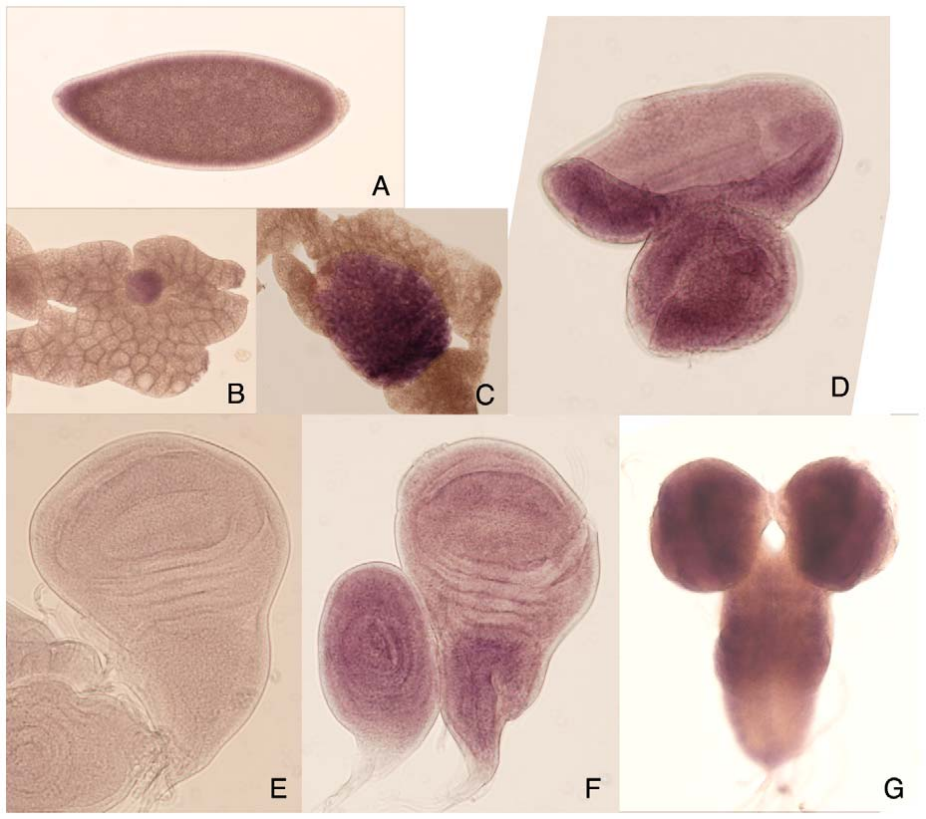
In situ hybridization by *Pngl* DIG-labeled RNA probes. (A) stage 4 embryo, which is the oldest stage of the embryonic positive staining; (B) larval ovary; (C) larval testis; (D) eye-antennal disc, dorsal to the left and anterior to the top; (E) control staining of wing disc by sense probe; (F) wing (and leg) disc, anterior to the left; (G) larval brains, dorsal view and anterior to the top.

To examine the spatio-temporal expression of *Pngl* during development, *in situ* hybridization was carried out using embryos or larval tissues. Strong expression of mRNA was only observed in early embryos up to stages 4 to 5, prior to the stage of cellularization (Fig. 3A). The high level of mRNA detected in very early embryonic stages is probably due to maternally provided transcripts expressed during oogenesis. Expression of somatic mRNA at later embryonic periods was not detected under the experimental conditions (data not shown). However, among the larval tissues distinct levels of mRNA expression was observed mainly in the gonads, both testis and ovary, and imaginal discs including eye, wing and leg discs, and central nervous system (Fig. 3B–D, F and G). The expression detected with *Pngl* antisense probes is specific as no signal was detected using DIG-labeled RNA probes prepared against the sense strand of cDNA (Fig. 3E). These results suggest that Pngl might play a critical role during larval development and metamorphosis.

### Homozygous deletion mutants show developmental defect

**Figure 4:**
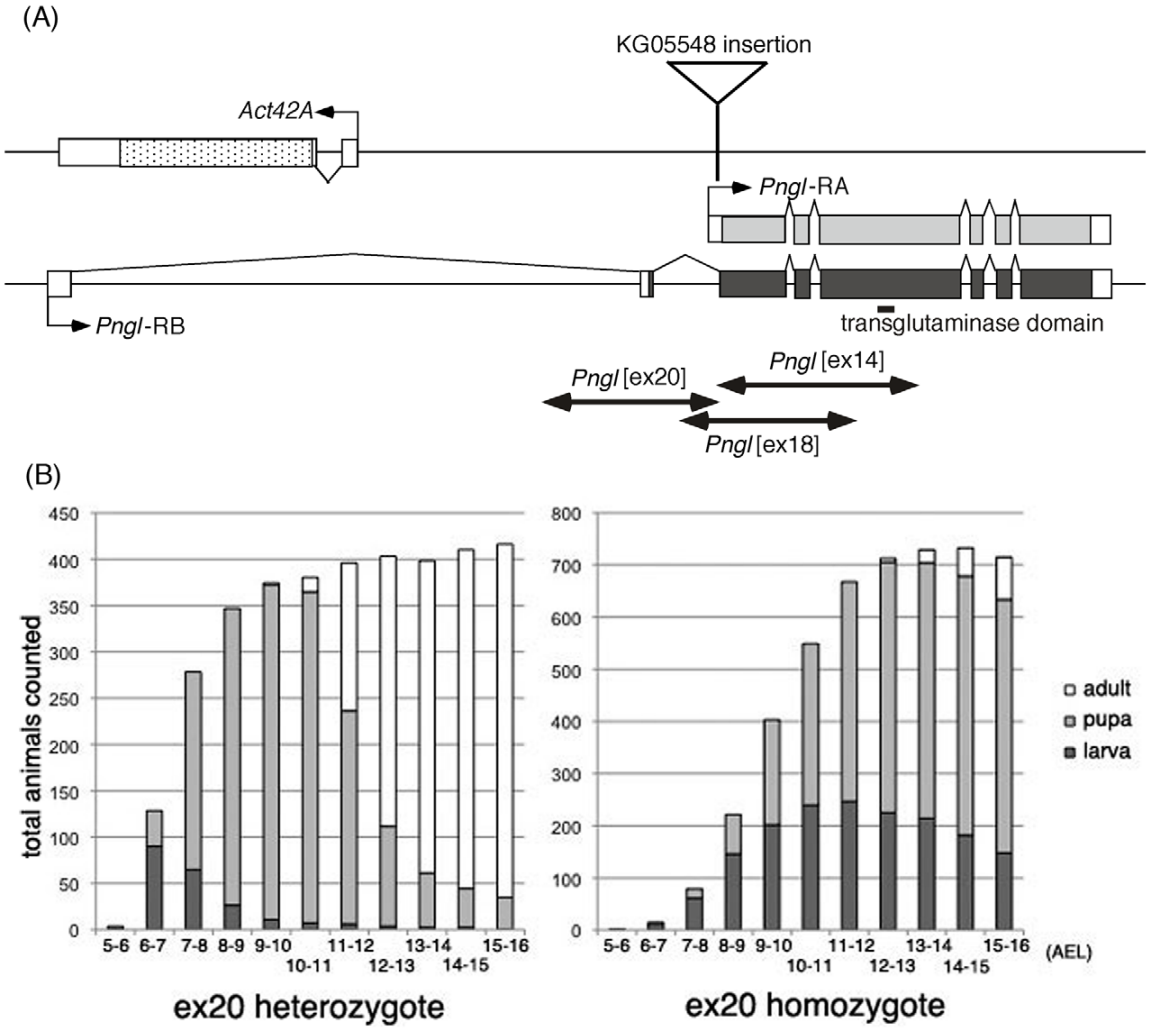
Mutation of *Pngl* resulted in severe delay in development. (A) genomic map of *Pngl* with the 2 predicted mRNA, *Pngl*-RA(light grey) and *Pngl* RB (dark grey), deletion points in ex14, 18 and 20, indicated by the arrows; (B) Quantitative analysis of *Drosophila* growth of heterozygotes and homozygotes of the *Pngl* microdeletion *Pngl*[ex20]. The number of days after egg laying (AEL) is plotted on the X axis and the number of animals counted is plotted on the Y axis. White indicates the adult, light grey, pupae and dark grey, wandering larvae.

To provide more insight into the biological significance of the cytoplasmic PNGase ortholog in flies, we generated and characterized mutations in the *Pngl* gene. Accordingly, imprecise excision of a P element transposon insertion in the *Pngl* locus, *Pngl*[KG], was carried out and 3 microdeletion strains, *Pngl*[ex14], *Pngl*[ex18] and *Pngl*[ex20] were isolated. The deletions were molecularly characterized by PCR with DNA primers flanking the deletion breakpoints and sequencing of the resulting PCR products. The *Drosophila* genome project identified two splice variants of *Pngl*, CG7865-RA (*Pngl*-RA) and CG7865-RB (*Pngl*-RB) that are predicted to encode almost identical proteins; RB encodes a protein with 7 amino acids shorter at the N-terminus (http://flybase.org/reports/FBgn0033050.html). The *Pngl*[ex14] and *Pngl*[ex18] deletions both extend from just upstream of the shorter *Pngl-RA* transcript (within the second intron of the longer *Pngl-RB* transcript) into the protein coding region (Fig. 4A), and in both cases are predicted to express in-frame truncated proteins by RT-PCR analysis. On the other hand, no transcripts could be detected from the *Pngl*[ex20] deletion which extends 1.1 kb upstream from *Pngl*[KG] transposon insertion site. This result indicates that *Pngl*[ex20] is a *bona fide* null allele for *Pngl*.

Having established mutant strains for Pngl, we next examined the phenotypes of them. All three deletions were semilethal, producing homozygous adults at less than 1% relative to their heterozygous siblings. This same phenotype was observed for flies heterozygous for the different deletions, and for flies heterozygous for the deletions and the chromosomal deficiency *Df(2R)nap* [9], clearly showing that these mutants were all allelic with similar semilethal phenotypes.

For a detailed analysis of the mutant phenotype, 1st instar larvae homozygous for *Pngl*[ex20] were separated from their heterozygous siblings and the growth curve was examined. Heterozygotes with the GFP balancer, collected from the same cross were also examined as the control. It was found that the homozygous larvae started to wander a couple of days later (Fig. 4B, right panel) than the heterozygotes (Fig. 4B, left panel), which showed the similar growth rate with that of the wild type (data not shown). Homozygotes also showed significant delay in the pupal formation, resulting from the extended larval period. Transheterozygotes between *Pngl*[ex14] and *Pngl*[ex20] were also examined and showed the same growth delay and semilethal phenotype as the *Pngl*[ex20] homozygotes (data not shown). Despite the delayed growth, several percent of the animal succeeded in pupal eclosion to emerge as adults without any apparent morphological abnormality. Nevertheless, the surviving adult flies exhibited a higher frequency of premature death than that of wild type, as well as severe male and female sterility; sterility was observed for 4 out of 5 *Pngl*[ex20] homozygous males, 2/5 of *Pngl*[ex14]/*Pngl*[ex20] transheterozygous males, 15/17 of *Pngl*[ex20] females and 17/18 of *Pngl*[ex14]/*Pngl*[ex20] females. These results indicate that a basal level expression of Pngl is critical for normal lifespan and reproduction process.

### Transgene expression of *Pngl* rescued phenotypic consequences of mutants

**Figure 5:**
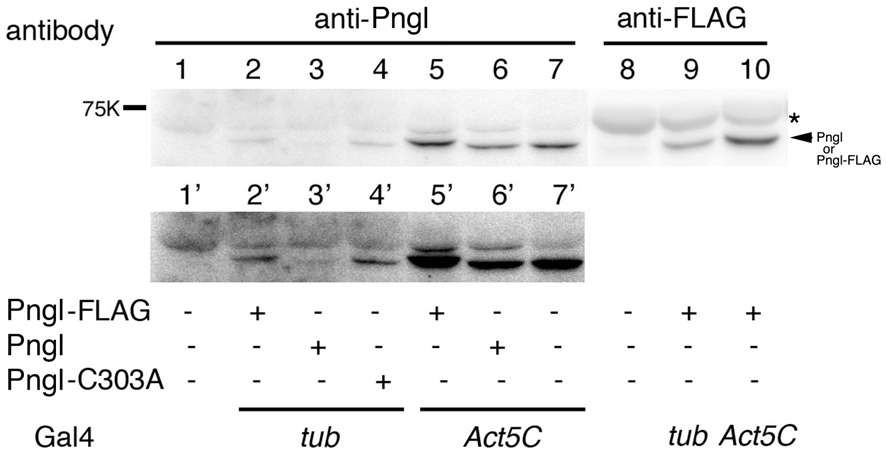
Detection of Pngl or Pngl(C303A) expressed by UAS-Gal4 system. By Western blotting, blots were stained with rabbit anti-Pngl antibody (lanes 1–7), or mouse anti-FLAG monoclonal antibody (M2, lanes 8–10). All samples were soluble fractions isolated from larvae. Lower panel (lanes 1′–7′) represents the longer exposure of the upper panel (Lanes 1–7). Lanes 1 and 8: yw larvae (wild type control); lanes 2 and 9: Pngl-FLAG driven by *tub*-Gal4; Lane 3: Pngl driven by *tub*-Gal4, lane 4: Pngl(C303A) driven by *tub*-Gal4; Lanes 5 and 10: Pngl-FLAG driven by *Act5C*-Gal4, Lane 6: Pngl driven by *Act5C*-Gal4, and Lane 7: Pngl(C303A) driven by *Act5C*-Gal4. The asterisk indicates the non-specific bands bound by Pngl peptide antibody. The band for Pngl or Pngl-FLAG protein is indicated by an arrow.

To unequivocally show that the observed phenotypes of the mutants were due to the lack of Pngl, we investigated the effect of *Pngl* transgene expression in these mutants. Expression of a UAS-*Pngl* construct driven either by *tub*-Gal4 or *Act5C*-Gal4, both of which result in ubiquitous expression, completely rescued the developmental delay and semilethal phenotype of the homozygous *Pngl* mutants (Table 2). This result clearly indicates that the phenotypic consequences of mutants were indeed due to the lack of *Pngl*. In addition, expression of mouse PNGase ortholog, Ngly1 was also found to rescue the semilethality (Table 2), strongly suggesting that the functional importance of Pngl is conserved among species. Ectopic expression of *Pngl* to specific tissues, so far as tested, failed to rescue the strong semilethal phenotypes, suggesting that Pngl functions in a cell autonomous fashion, and not by secretion or transcytosis from surrounding cells. Importantly, while the wild type Pngl and FLAG-tagged Pngl rescued the semilethality in a similar manner, the Pngl(C303A) mutant, in which Cys serving as catalytic residue in other orthologs was changed to Ala, could not rescue the growth delay and semilethal phenotype either with *tub*-Gal4 or *Act5C*-Gal4 driver line, despite the equivalent level of protein expression (Fig. 5). These results strongly suggest that the transglutaminase domain of Pngl participates in some type of catalytic activity even though the protein does not have the conventional deglycosylation activity.

**Table 2:** Rescuing abilities of each UAS-Gal4 combination.

Gal4	Expression patterns	UAS construct			
		*Pngl*	*Pngl*-FLAG	*Pngl*, C303A	*Ngly1*
*tub*-Gal4	Ubiquitous (weak)	+	+	−	+
*Act5C*-Gal4	Ubiquitous (strong)	+	+	−	+
*vg*-Gal4	A part of disc	−	−	−	n.d.
GMR-Gal4	Eye disc	−	−	−	n.d.
c855a-Gal4	outer proliferation center of the larval optic lobe	−	−	−	n.d.

+, rescued; − no rescue observed; n.d., not determined.

## Discussion

Although recent studies have established the involvement of the cytoplasmic PNGase in ERAD-related events, how this enzyme activity may relate to its biological significance has not been demonstrated in any systems. In this study, we report the surprising finding that, although biologically shown to be critical for normal development, the functional importance of the fly PNGase ortholog (Pngl) may not lie in its deglycosylation activity.

The cytosolic PNGase is an intriguing protein as, during the evolutional process, apparent orthologs have resulted in quite diverse structural organization between species. For example, *C. elegans* PNGase also has thioredoxin-like domains with oxidoreductase activity thus serving as a dual functional enzyme [38], [39], and mammalian PNGase has the so-called PUB domain at its N-terminus, which can play a role as a protein-protein interaction domain [25], [40], [41], [42], [43]. At the C terminus the putative mannose-binding domain may contribute to the interaction with *N*-linked glycans [19], [21] (Fig. 4). On the other hand, *Arabidopsis* PNGase (AtPng1) is quite distinct with totally different domains both N- and C-terminal of the transglutaminase/PNGase domain (see Fig. 1A). There is a report that AtPng1, unlike yeast Png1, possesses a transglutaminase activity [44] in addition to its PNGase activity [30]. Indeed, this structural diversity may result in diverse functions depending on the organisms. It is important to note here that the overall structure of Pngl and mammalian orthologs are quite similar [9], implicating similar molecular functions between them. Indeed, the protein was found to retain its carbohydrate-binding activity as in the case of PNGases from mammal, which was previously reported to be important for the enzymatic activity [21]. As the binding was highly specific to *N*-glycan derivatives containing *N,N′*-diacetylchitobiose structure [31], it is likely that the interaction between the *N*-linked glycans and the PNGase ortholog is a conserved phenomena.

It is also interesting to note that, through use of systematic high-throughput analysis, Pngl was also shown to interact with Rad23 ortholog in the fly [45], as were the orthologs in other species [12], [40]. We also confirmed the binding between those proteins by the yeast two-hybrid assay (data not shown). These results also suggest the conservation of its molecular functions throughout evolution.

There is, however, also a quite distinct structural feature in Pngl, as it lacks the second CXXC motif, which forms a zinc-binding domain critical for enzymatic activity [18], [22]. Consistent with this finding, we could not detect any PNGase activity by either *in vitro* or *in vivo* assays. Interestingly, the second CXXC motif, while lost among many of the Diptera, is still conserved in some other insects, such as *Apis mellifera* and *Tribolium castaneum* (Fig. 2A). Therefore one could envisage that the loss of the second CXXC motif most likely occurred within the insect species, but it did not affect the viability of these species although the enzyme activity may have been lost.

The fact that the mutation in this gene can lead to failure of proper development demonstrates the functional importance of Pngl. It is interesting to note that the expression levels of PNGase may not be critical as ubiquitous *Pngl* expression did not produce adverse effects, even when the endogenous expression levels were observed to be under specific spatio-temporal control. It should also be noted that the biological significance of this protein does not seem to lie in the deglycosylation activity, because Pngl lacks the second CXXC motif critical for PNGase activity. It is therefore possible that, given the strikingly similar domain organization, mammalian ortholog of the cytoplasmic PNGase, besides its PNGase activity, may also exert its functions through the similar deglycosylation-independent mechanism, and indeed it was suggested by our observation that the defect of *Pngl* mutation was rescued by the expression of Ngly1, a mouse ortholog of Pngl (Table 2).

Interestingly, as shown by the failure in the rescue by the expression of Pngl(C303A) mutant, in which Cys serving as catalytic residue in other orthologs was changed to Ala (Table 2), this cysteine, may still have important roles for the proper function of the protein, if not for a PNGase activity. An alternative explanation could be that the Cys to Ala mutation resulted in conformational change thereby causing premature degradation, although this seems unlikely as the mutant protein is expressed well in insect cells (cf. Fig. 3B) and *in vivo* (Fig. 5).

PNGase ortholog NcPNG1 from *Neurospora crassa* also was recently reported for the loss of PNGase activity. However, the loss occurred mainly due to the evolutional amino acid change in the ‘catalytic triad’ in the domain corresponding to the active center. It was also shown that the PNGase activity is apparently not needed as the catalytic-inactive C to A mutation of ScPng1 from *Saccharomyces cerevisiae* rescued the hyphal extention-failure phenotype [24]. Situation therefore is quite distinct from the case with Pngl, where the equivalent C-to-A mutation could not rescue the developmental defect (Table 2). Interestingly, the semilethal phenotype of *Pngl* excision was rescued by the mouse Ngly1 (Table 2), strongly suggesting that certain biochemical property, which is inevitable for the fruitfly viability, is also conserved among species.

To examine the molecular detail of its defect, we examined the pattern of cell cycle marker such as BrdU staining or that of ecdysone receptor subtypes (EcR-A, 15G1a; EcR-B1, AD4.4; or EcR-common, Ag10.2) in the mutant tissues, but no apparent abnormality was observed (data not shown). Thus the detailed biochemical properties still remain to be determined.

In summary, we characterized a *Drosophila* PNGase ortholog, which produces a protein product that lacks conventional PNGase activity, but nonetheless plays important roles during larval and pupal development as well as contributing to the normal longevity and fertility of adults. Now that functional diversification of this protein is evident, efforts should be directed to clarify the precise molecular functions of this protein from various organisms. Although Pngl appears to have lost the PNGase activity, the putative “catalytic” Cys was still shown to be important for its function. Although the contribution of each domain such as the PUB domain or the mannose-binding domain remains unknown, the fact that the point mutation to the “catalytic” Cys resulted in loss of functionality suggests that some enzymatic function of this protein may be required for its proper function. Whatever the biochemical activity, this report indicates that unlike the case in yeast, mutations in the *Drosophila* ortholog of cytoplasmic PNGase can cause severe developmental phenotypes, thereby indicating an unclarified biological role for this protein in higher eukaryotes, especially the mammalian ones with the similar domain structure. Considering the quite distinct domain organization of different PNGase orthologs, this protein could also serve as an intriguing model to determine how one protein acquired such diverse structures during evolution and understand how the distinct biochemical properties of these proteins contribute to their functional importance.

## Materials and Methods

### Expression of the Drosophila PNGase ortholog (Pngl) in S2 cells

The cDNA clone SD19435 from the *Drosophila* Gene Collection encoding the putative *Drosophila* PNGase ortholog [46] kindly provided by Dr. Ryu Ueda at National Institute of Genetics, Mishima, was used as the template for preparing various constructs. Primers used for PCR amplification of Pngl ORF are listed in Table 3. Amplified DNA was cloned into the plasmid pRmHa using *Bgl*II and *Xho*I sites to replace the insert of pRmHa-OFutI (a gift from Drs. Ken Irvine and Tetsuya Okajima, Rutgers University) producing pRmHa-*Pngl*. This construct contains a (His)_6_-tag at the C terminus of Pngl and expression is under the control of the inducible metallothionein promoter. The integrity of the construct was confirmed by sequencing using BigDye ver 3.1 and the 3130xl DNA sequencer (ABI, CA). The construct was transfected into *Drosophila* S2 cells using Cellufectin (Life Technologies, CA) according to the manufacturer's protocol. Protein expression was induced by adding CuSO_4_ solution (final 0.7 mM) into the culture medium for 72 hours [47] and the cells were harvested. (His)_6_-tagged Pngl protein was identified by Western blotting using anti-His antibody (Santa Cruz Biotechnology, CA).

**Table 3:** Summary of primers used in this work.

Primers for vector construction		
pRmHA-*Pngl* (*Bgl*II-*Xho*I)	F	GAAGATCTATGTGGCAGCTGGTCATC
	R	CCGCTCGAGATGTAGTTGCACTTGCAGATCGAAGG
pVL1393-*Pngl* (*Not*I-*Xba*I)	F	ATAAGAATGCGGCCGCCTAAAAATGTGGCAGCTGG
	R	CGCGGATCCCTACTTATCGTCGTCATCCTTGTAATCATGTAGTTGCACTTGCAGATCGAA(with FLAG)CGCGGATCCTCAATGTAGTTGCACTTGCAGATCGAA (without FLAG-tag)
pVL1393-*Ngly1*-FLAG (*Eco*RI-*Eco*RI)	F	CGGAATTCGCCACCATGGCGTCGGCCAC
	R	CGGAATTCCTACTTATCGTCGTCATCCTTGTAATCGAGGTCATTGAACGTTATAAT
pUASP-*Pngl* (*Not*I-*Bam*HI)	F	ATAAGAATGCGGCCGCCTAAAAATGTGGCAGCTGG
	R	CGCGGATCCCTACTTATCGTCGTCATCCTTGTAATCATGTAGTTGCACTTGCAGATCGAA(with FLAG)CGCGGATCCTCAATGTAGTTGCACTTGCAGATCGAA
pUASP-*Ngly1* (*Ngly1* ORF)	FR	TTCATTGGTACCCGCGCCACCATGGCGTCGGCCACTCGAGGTCGACTCTATCAGAGGTCATTGAACGTTATAAT

primers for mutagenesis		
Pngl(C303A)	F	TAAGGGCCGTGCCGGAGAATATGCCAATTG
	R	GGCATATTCTCCGGCACGGCCCTTACGGGA
ScPng1(TVCC)	F	GAGATCTATAAAACCGTGTGCTGCGGAAATATCAC
	R	GATATTTCCGCAGCACACGGTTTTATAGATCTCAACAG
ScPng1(TNRC)	F	GATCTATAAATGCGTCTGTTGCGGAAATATCACTAGATTTC
	R	CCGCATCGGTTCGTTTTATAGATCTCAACAGTTC
ScPng1(CVCC)	F	GATCTATAAATGCGTCTGTTGCGGAAATATCACTAGATTTC
	R	GTGATATTTCCGCAACAGACGCATTTATAGATCTCAACAGTT

F, forward primer; R, reverse primer.

### Analaysis of PNGase activity in *Drosophila melanogaster* larval soluble fractions

Harvested 3rd instar wandering larvae or freshly dissected mouse testis were homogenized on ice in 2 vol (v/w) of 10 mM HEPES buffer with 250 mM sucrose, 2 mM DTT, 1mM AEBSF and 1×Complete protease inhibitor cocktail and ultracentrifuged at 100,000×*g* at 4°C for 1 hour. Then the soluble fractions obtained were transferred to new tubes and used as enzyme sources for the analysis of PNGase activity. The enzyme assay was ^14^C-labeled asialofetuin glycopeptide I as a substrate ([6], [37]. 10 µl reaction contains 1 µl of the substrate (14,000 cpm), 6 µl of the enzyme sources and 3 µl of various buffers (100 mM MES-NaOH pH 6.7, 100 mM HEPES-NaOH pH 7.4 or 100 mM HEPES-NaOH pH 8.3). Reaction was conducted at ambient temperature for overnight. The resultant deglycosylated peptide by the action of PNGase activity was separated by paper chromatography with buthanol/ethanol/water (2∶1∶1) and analyzed by BAS 2500 (Fujifilm, Tokyo, Japan). The soluble fractions isolated from wild type yeast PNGase (ScPng1) and its catalytic inactive form (ScPng1C191A) were used as positive or negative control for the enzyme reaction [8], [22].

### Protein expression in Sf21 cells

cDNA including a preceding possible KOZAK sequence at the 5′ end of the gene was amplified with the *Not*I and *Xba*I site at each end of the fragment using the primers listed in Table 3. The insert was ligated to pVL1393 plasmid (BD Biosciences, CA) after digestion with the appropriate enzymes to generate pVL1393-*Pngl*. For the production of C303A mutated Pngl (Pngl(C303A)), mutagenesis was conducted on the TA-cloned cDNA, which was amplified with the same PCR primers as above and subcloned into the pVL1393 vector by the same strategy. As a positive control for the glycan-binding experiment, mouse PNGase, Ngly1, was also cloned using the primers listed in Table 3 to produce *Eco*RI sites at the both ends and with a 3′ end FLAG-tag. After the restriction enzyme digestion, the *Ngly1* ORF-containing fragment was subcloned into pVL1393, which we denote ‘pVL1393-*Ngly1*-FLAG’. pVL1393 insertion constructs were transfected into Sf21 cells with BaculoGold DNA (BD Biosciences, CA) by Cellufectin (Life Technologies, CA) according to the manufactures' protocols. After the 3rd infection, cells were harvested by centrifugation, followed by washing with PBS. Cells were then homogenized with a cell grinder in buffer containing 10 mM HEPES-NaOH pH 7.4, 250 mM sucrose, 2 mM DTT, 1 mM AEBSF, and 1×Complete Protease Inhibitor Cocktail (Roche Diagnostics GmBH, Mannheim) and the supernatant was collected for enzyme assays after ultracentrifugation at 100,000×*g* for 1 hour at 4°C. Ngly1 activity was confirmed in PNGase assays using RNaseB as substrate [37].

### Generation of anti-Pngl antibody

The peptide containing the C-terminal sequence of Pngl, (C)RQSLNSRDYPFDLQ was synthesized and conjugated to KLH for use as an immunogen. Affinity-purified serum against the peptide was generated by Gene Design Inc. (Ibaraki, Japan). The specificity of the antibody was confirmed by the Western blotting with BSA-conjugated peptide and Sf21-expressed Pngl.

### Western blotting

The enzyme sources obtained from Sf21 cells were subjected to SDS-PAGE and Western blotting as described previously [48]. In brief, blotting was done by the submarine-type mini-transblot (Bio-Rad Laboratories, Tokyo) onto PVDF membrane (PALL, FL) and the blots were treated with 5% skim milk (Nacalai Tesque, Kyoto) for blocking. After the treatment of the membrane with the 1st antibody or HRP-labeled 2nd antibody dissolved in 5% skim milk protein was detected using the Immobilon Western HRP substrate (Millipore, MA) and LAS3000 mini (Fujifilm, Tokyo).

### Iodoacetamide glycan binding assay

The molecular properties of iodoacetamide *N*-glycan and the procedure of binding assay using this material were described previously [31]. Briefly, the supernatant after ultracentrifugation of homogenate from Sf21 transfected with pVL1393-*Pngl*-FLAG, *Pngl*-C303A-FLAG, or *Ngly1*-FLAG was mixed with 50 µM of Man_8_GlcNAc_2_-IAc and incubated for 10 min at 25°C. Then the mixture was subjected to SDS-PAGE and the proteins bound to the probe were visualized by Western blotting using anti-FLAG antibody M2 (Sigma-Aldrich, MO).

### Isolation and analysis of free oligosaccharides from larval soluble fractions

Free oligosaccharides (fOSs) were isolated from the soluble fractions of wild type- or mutant larvae. The fOSs isolated was labeled with 2-amino pyridine, and quantitated using the HPLC as described before [48], [49]. Detailed method are described in *Supplemental Information Materials and Methods* (Text S1).

### *In situ* hybridization

DIG-labeled RNA probes were produced by *in vitro* transcription using T7 (for control sense probe) or Sp6 RNA polymerase (for antisense probe) (Roche Diagnostics GmBH, Mannheim) using SD19435 plasmid digested with *Xho*I or *Eco*RI, respectively. *In situ* hybridization was conducted as described previously [50]. The stained samples were mounted in PBS containing 80% glycerol.

### Isolation of *Pngl*-deletion mutants

Deletions within *Pngl* were obtained via imprecise excision of the *P{SUPor-P}PNGase*[KG05548] P-transposon insertion, hereafter referred to as *Pngl*[KG]. [51], [52], which has an insertion at the proximity of ORF 5′ (http://flybase.org/reports/FBti0024510.html). The structure of each deletion allele was determined by sequencing amplified DNA fragments obtained by genomic PCR with primers flanking the deletion breakpoints.

Flies homozygous for the parental *Pngl*[KG] chromosome are inviable, but flies heterozygous for the *Pngl*[KG] chromosome and *Df(2R)nap* [9], a deficiency chromosome for this region are viable. These results indicate the presence of an extraneous lethal mutation on the parental *Pngl*[KG] chromosome. The *Pngl*[KG] transposon was mobilized using *CyO, H[Δ2-3]*. Flies of the genotype *y w ; Pngl*[KG] / *CyO, H[Δ2-3]* were mated to *y w ; al dp b cu px sp / CyO* and putative excision alleles were recovered as white-eyed, *CyO* male progeny that were back-crossed to *y w / y w ; al dp b cu px sp / CyO* females in order to generate balanced stocks. P-mobilization crosses done in the male germline resulted in the recovery of putative excision alleles from 22 of 24 independent crosses. Crosses done in the female germline produced putative excision alleles in 14 of 29 crosses. All three of the confirmed deletion alleles of *Pngl* were obtained via mobilization in the male germline. These three deletions, as well as 23 of the other excision alleles were all homozygous lethal and lethal over the parental *Pngl*[KG] chromosome due to the extraneous lethal. Meiotic recombination was used to remove this lethal mutation from the *Pngl*[ex14.1] (*Pngl*[ex14]), *Pngl*[ex18.3] (*Pngl*[ex18]) and *Pngl*[ex20.5] (*Pngl*[ex20]) chromosomes used in this work. Each of these chromosomes is fully viable over the parental *Pngl*[KG] chromosome and shows reduced viability over *Df(2R)nap* [9]. Again, deletions were confirmed by genomic PCR.

### The production of UAS-*Pngl* flies

The ORF of the cDNA, including a putative KOZAK sequence, with additional restriction enzyme sites for cloning were amplified with the primers listed in Table 3, thereby producing *Not*I or *Bam*HI sites at its 5′ or 3′ end, respectively. The amplified fragment was then cloned into TOPO-TA vector (Life Technologies, CA) according to the manufacturer's protocol to generate TA-*Pngl* plasmid. The insertion was isolated by the digestion with *Not*I/*Bam*HI, and cloned into the pUASP vector [53]. For the addition of 3′ FLAG-tag, a different primer was used as the reverse primer (listed in Table 3), and was cloned into pUASP as described above. Mutagenesis of the cysteine residue in the active site was done using the TA-*Pngl* plasmid with the primers listed in Table 3. The insert was cloned into the pUASP vector as described above. The pUASP-*Pngl*, pUASP-*Pngl*-FLAG and pUASP-*Pngl*(C303A) DNA constructs were sent to Genetic Services Inc. for injection to establish transgenic *Drosophila* lines.

### Preparation of UAS-*Ngly1* strains for the rescuing *Pngl* by homologous protein

A full length of ORF of *Ngly1* was amplified by the primers shown in Table 3 to add a short fragment of pUASP at the 5′ and 3′, allowing the recombination into pUASP vector by In-Fusion Advantage Cloning Kit (Clontech Laboratories, Inc., CA). The insert was confirmed by sequencing. The plasmid pUASP-*Ngly1* was sent to Best Gene Inc. for the injection to produce transgenics.

### Site-directed mutagenesis and PNGase activity assay of ScPng1

Site-directed mutagenesis of (His)_6_-tagged ScPng1 in pET28b [8] was carried out essentially as described previously [22] using pET28b-ScPng1 as a template. Primers used are listed in Table 3. PNGase activity was examined as described previously [37]. Briefly, the substrate, *S*-alkylated RNaseB was mixed with the bacterial extract [8] expressing ScPng1 or its derivative mutants. The activity was analyzed by the amount of deglycosylated substrate separated from the substrate by SDS-PAGE.

### Genotypes of flies used

y w, and Canton S were used as wildtype controls. The fly strains with following genotypes were generated by crosses:

*Pngl*/ CyO Act-GFP
*Pngl vg*-Gal4/ CyO
*Pngl Act5C*-Gal4/ CyO
*Pngl*/ CyO; *tub*-Gal4
*Pngl* GMR-Gal4/ CyO
*Pngl*/ CyO; c855a-Gal4
*Pngl* UAS-*Pngl*/ CyO
*Pngl* UAS-*Pngl*-FLAG/ CyO
*Pngl* UAS-*Pngl*(C303A)/ CyO
*Pngl* UAS-*Ngly1*/ CyO

KG08854 and c855a were obtained from the Bloomington Stock Center (Indiana Univ.), a double balancer stock used for generating different *Drosophila* stocks was from the *Drosophila* Genetics Resource Center at Kyoto (Kyoto Institute of Technology). The CyO-GFP balancer, *vg*-Gal4, *tub*-Gal4, and GMR-Gal4 stocks were originally from the Kyoto Stock Center, and the *Act5C*-Gal4 stock [54] was kindly provided by Dr. Shoko Nishihara (Soka University, Japan).

### Growth analysis

One hundred 1st instar larvae were selected based on the presence (heterozygotes) or absence (homozygotes or transheterozygotes) of the GFP-expressing balancer chromosome. The numbers of wandering larvae, pupae, or adults were counted everyday.

### Rescue experiment

PNGase deletions were recombined onto the *Act5C*-Gal4, UAS-*Pngl* or UAS-*Ngly1* chromosome to generate *Act5C*-Gal4 *Pngl*[ex], UAS-*Pngl Pngl*[ex], or UAS-*Ngly1 Pngl*[ex]. For expression by *tub*-Gal4, *Pngl*[ex] / CyO; *tub*-Gal4/ MKRS flies were prepared. These flies were crossed to determine if the progeny contain flies of the genotype *Act5C*-Gal4 *Pngl*[ex] / UAS-*Pngl* or UAS-*Ngly1 Pngl*[ex], or *Pngl*[ex] / UAS-*Pngl Pngl*[ex]; *tub*-Gal4 /+.

## Supporting Information

Text S1 Evidence for an essential deglycosylation-independent activity of PNGase in *Drosophila melanogaster*.
(0.07 MB DOC)

Table S1 The fOS species predicted in the soluble fractions obtained from wild type or Pngl mutant 3rd instar larvae.
(0.23 MB DOC)
