# Supplementary material for: Evidence for an Essential Deglycosylation-Independent Activity of PNGase in Drosophila melanogaster
Source: PLoS One. 2010 May 10;5(5):e10545. doi: 10.1371/journal.pone.0010545 (PMC2866665; doi:10.1371/journal.pone.0010545)
Supplement: Text S1 — Evidence for an essential deglycosylation-independent activity of PNGase in Drosophila melanogaster. (0.07 MB DOC) [file pone.0010545.s001.doc]

**Text S1**

**Evidence for an essential deglycosylation-independent activity of PNGase in *Drosophila melanogaster***

**Purification and analysis of neutral free oligosaccharides (fOSs) from larval soluble fractions**

**Materials and Methods**

Twenty wandering larvae with the size of normal wandering larva were homogenized in 40 μl of extraction buffer (10 mM HEPES, 1 mM EDTA, 0.25M Mannitol, 2 mM DTT, 1 mM AEBSF and 1 x Complete Protease Inhibitor Cocktail (Roche Diagnostics GmBH, Mannheim)) and the extract was subjected to the ultracentrifugation at 100,000 x *g* for 1 hour at 4ºC. The supernatant thus obtained was mixed with the same volume of chloroform/ methanol (2:1) for delipidation and deproteination. The free oligosaccharide in the aqueous solution was precipitated by adding 1.5 vol of ethanol (final 60%) and the supernatant fractions containing fOSs were collected by centrifugation at 14,000 rpm for 10 min at 4ºC. The supernatant was evaporated to reduce the ethanol content to less than 40% and was applied onto AG50w resin (H^+^ form, 500 μl of bed volume) and AG1-X2 resin (acetate form, 500 μl of bed volume) (Bio-Rad, Hercules, CA) for deionization. The flowthrough fraction containing neutral fOSs was desalted with PD-10 column (GE Healthcare Biosciences AB, Uppsala; equilibrated with 5% ethanol) according to the manufacturer’s instruction and was evaporated to dryness. Pyridylamination (PA) of the free oligosaccharide was carried out as previously described [1,2].

The unreacted PA in the reaction mixture was removed using a monolithicsilica spin column (MonoFas® (GL-Science)), as described previously [2]. The PA-labeled free oligosaccharide mixture was then digested with excessive amount of glucoamylase (Wako Pure Chemical Industries, Inc., Osaka, Japan) in pH 5.0 of 50 mM acetate buffer at 40ºC for 16 hours and the reaction was stopped by the heat denature (100ºC for 10 min.) followed by the addition of 1.5 vol. ethanol. The supernatant was collected by centrifugation and the supernatant was dried up, dissolved in dH_2_O and was subjected to the precise structural analysis using HPLC system as follows. To note, the detailed analysis was done on the mixture of fOS fraction from 3 batches of larvae because of the limited amount of the fOSs.

Separation of free oligosaccharides by the size-fractionation HPLC on Shodex NH2P-50 4E column (0.46 mm i.d. x 250 mm, Shodex, Tokyo, Japan) was carried out as described previously [1,2]. After the size fractionation, the fOSs obtained was further separated using the reversed-phase HPLC, an Inertsil ODS-3 column (2.1 mm i.d. x 150 mm, GL Sciences, Tokyo, Japan), was used as described previously [3]. Among the peaks detected, the high mannose-type glycans were identified by excessive amount of Jack bean α-mannosidase treatment (40 mU/20 μl reaction) (Seikagaku Corp., Tokyo, Japan) in 50 mM sodium acetate buffer, pH 5.0 at 37ºC for 16 hours. The structures of OSs were identified using the glucose unit (GU) of reversed-phase HPLC as previously reported [3].

**Results and Discussions**

Structures of high mannose-type fOSs in the soluble fraction isolated from the homogenate of wild type (Canton-S) or *Pngl*[ex20] homozygous wandering larvae was examined by HPLC, and average amount obtained is shown in Table S1. During this experiment, we noted that the amount of fOSs was found to fluctuate largely among the samples (data not shown). In some batches it was found that amount of fOSs from *Pngl*[ex20] was observed to be even higher than the one obtained from wild type.

It needs to be noted that due to the elongated and variable length of larval period for the *Pngl* mutants resulted from the growth delay, it was not possible to collect the *Pngl* larvae with precisely the same physiological condition with that of the wild type. To say the least, we could not provide convincing evidence based on the fOS analysis that the Pngl has a *bona fide* PNGase activity. Furthermore, loss of one of the CXXC motifs shown to be critical for the deglycosylation activity in Pngl made the possibility of PNGase activity in Pngl protein unlikely (see text).

Interestingly, the major possible high-mannose *N*-linked glycan is predicted to be M5A or M5A’ species, which was a similar result reported in the case of *Caenorhabiditis elegans* [4], not like in the case of mammalian cytosolic fOSs, whose major structure is M5B’ isoform [1,5]. Previously, we did not find apparent ortholog of Man2C1, which is a mammalian cytosolic α-mannosidase and did not observe any significant mannosidase activities at the neutral pH in the soluble fraction obtained from larval homogenate (data not shown), despite the occurrence of 8 apparent Man2B1 homologs with the unknown functions [6]. Thus it is therefore possible to assume that flies and worms may share similar processing mechanism for fOSs, irrespective of their source.

It is also noted that the fOSs isolated from fruitflies contain the Gn1 species (OSs with one GlcNAc at the reducing end), which implies the possible cytosolic endo-β-*N*-Acetylglucosaminidase (ENGase) activity as reported in other species [4,7]. This enzyme was shown to cleave the glycoside bond in the *N, N’-*diacetylchitobiose structure at the reducing end of *N*-linked glycans to generate the Gn1 fOSs. Consistent with this observation, occurrence of the ortholog for this gene was already predicted in *Drosophila melanogaster* ([7], CG5613: http://flybase.org/reports/FBgn0030839.html).

**References:**

1. Suzuki T, Hara I, Nakano M, Shigeta M, Nakagawa T, et al. (2006) Man2C1, an α-mannosidase, is involved in the trimming of free oligosaccharides in the cytosol. Biochem J 400: 33-41.

2. Hirayama H, Seino J, Kitajima T, Jigami Y, Suzuki T (2010) Free oligosaccharides to monitor glycoprotein endoplasmic reticulum-associated degradation in *Saccharomyces cerevisiae*. J Biol Chem *in press*.

3. Suzuki T, Matsuo I, Totani K, Funayama S, Seino J, et al. (2008) Dual-gradient high-performance liquid chromatography for identification of cytosolic high-mannose-type free glycans. Anal Biochem 381: 224-232.

4. Kato T, Kitamura K, Maeda M, Kimura Y, Katayama T, et al. (2007) Free oligosaccharides in the cytosol of Caenorhabditis elegans are generated through endoplasmic reticulum-golgi trafficking. J Biol Chem 282: 22080-22088.

5. Ohashi S, Iwai K, Mega T, Hase S (1999) Quantitation and isomeric structure analysis of free oligosaccharides present in the cytosol fraction of mouse liver: detection of a free disialobiantennary oligosaccharide and glucosylated oligomannosides. J Biochem (Tokyo) 126: 852-858.

6. Suzuki T (2009) Introduction to “Glycometabolome”. Trends in Glycoscience and Glycotechnology 21: 219-227.

7. Suzuki T, Yano K, Sugimoto S, Kitajima K, Lennarz WJ, et al. (2002) Endo-β-*N*-acetylglucosaminidase, an enzyme involved in processing of free oligosaccharides in the cytosol. Proc Natl Acad Sci U S A 99: 9691-9696.
