# Supplementary material for: Evidence for an Essential Deglycosylation-Independent Activity of PNGase in Drosophila melanogaster
Source: PLoS One. 2010 May 10;5(5):e10545. doi: 10.1371/journal.pone.0010545 (PMC2866665; doi:10.1371/journal.pone.0010545)
Supplement: Table S1 — The fOS species predicted in the soluble fractions obtained from wild type or Pngl mutant 3rd instar larvae. (0.23 MB DOC) [file pone.0010545.s002.doc]

| Gn2 | possible OS structures | Canton-S  (pmol/  100 larvae) | *Pngl*[ex20]  (pmol/  100 larvae) |
| --- | --- | --- | --- |
| M5A | 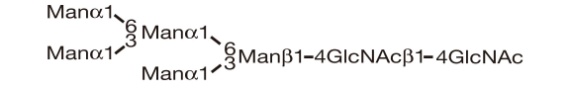 | 4.72 | 3.00 |
| M6B | 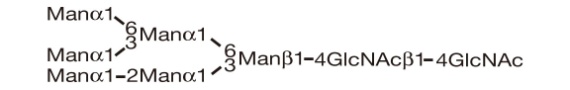 | 1.49 | 1.70 |
| M6C | 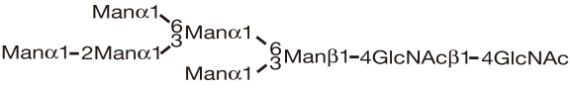 | 0.17 | n. d. |
| M7A | 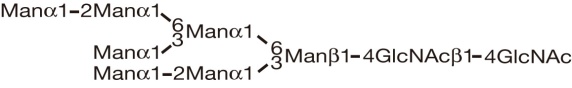 | 0.40 | n. d. |
| M7B | 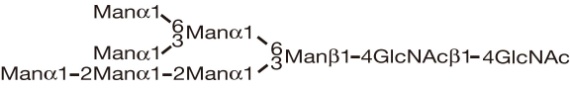 | 1.54 | n. d. |
| M8A | 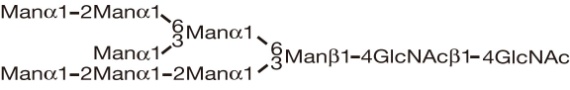 | 1.20 | 0.26 |
| M8C | 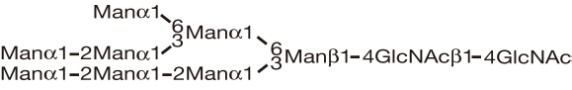 | 0.82 | 0.41 |
| M9A | 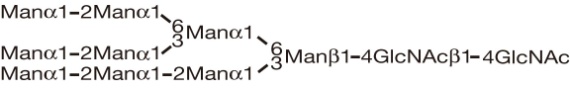 | n. d. | 0.25 |
| Gn1 |  |  |  |
| M5A' | 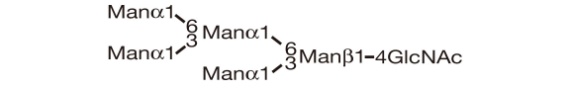 | 5.38 | 3.73 |
| M6B' | 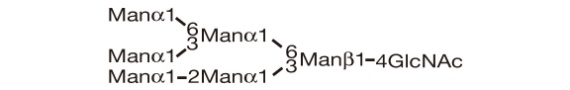 | 0.48 | n. d. |
| M6C' | 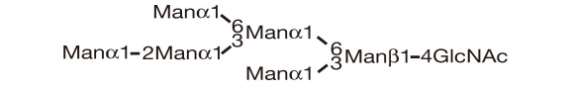 | 0.51 | n. d. |
| M7B' | 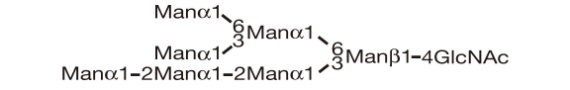 | 1.10 | 0.76 |
| M8A' M8B' M9A' | 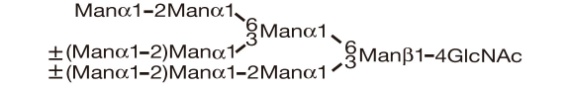 | 1.47 | 1.92 |
| M8C' | 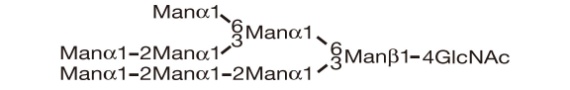 | 0.76 | n.d. |

n.d., not detected
